# Our New York Letter

**Published:** 1889-05

**Authors:** M. B. Hutchins

**Affiliations:** New York


### Correspondence.

### OUR NEW YORK LETTER.

### GYNECOLOGY.

At the Academy of Medicine, on the evening of the 4th inst., Dr. Thomas read a paper on "Acute Melancholia, Hypochondriasis and Mania, following operations upon Women." Gave details of six cases in his own experience which developed one of these symptoms, or all in succession, in from a few days to three months after the operation. Laparotomy, perineorrhaphy and operations upon the breast and uterus had been succeeded by the symptoms. Four of his six cases died—after symptoms of melancholia and acute mania developed—usually in coma. Had been able to collect from literature twenty-two cases, the symptoms of which entitled them to a place under the title given. During the discussion which followed, eleven similar cases were mentioned. Two of Dr. Thomas' cases gradually recovered.

Symptoms of the reported cases could not be attributed to septicæmia, iodoform or carbolic poisoning, nor considered as due to ether. In the discussion much was said with regard to the occasional poisonous effects of iodoform. Tendency to melancholia was thought sufficient ground for declining to operate. Long anticipation of the operation, and fear of death under its performance, thought to be predisposing causes of the bad symptoms in some cases.

One gentleman wandered from the subject, and mentioned cases relieved of mental aberrations and melancholia by undergoing operations for cure of the exciting cause. The President

(Dr. Loomis) mentioned by permission of Dr. T., a case of his own, in which symptoms similar to those described, had followed removal of a kidney. In closing, Dr. Thomas said if there was the slightest evidence that use of iodoform was ever followed by these symptoms, we would be justified in never using it. Mentioning the disagreeable properties of ether, he expressed the hope that the profession would once more give chloroform a trial. This sentiment was applauded. The profession abroad, he said, had never used ether so much as it is used here.

The woman whose uterus was removed, as described in my last letter, made a good recovery, and has returned home much improved in health. As the disease had involved the posterior vaginal wall, there can be only a question of time as to a return of her symptoms, and the usual termination of the disease, but at present, the operation is justified by the relief afforded.

Apiol is much used in the cases of amennorrhœa, which I have seen, and frequently with benefit.

### DERMATOLOGICAL NOTES.

Purpura, or peliosis rheumatica, is so rare, comparatively, that the description of the following case will be justified: Patient about twenty-eight years old. Trouble present since January, beginning with rheumatic pains. Previous health good, no former attack of rheumatism. "Cramps" occurrred in limbs and eruptions appeared on surfaces between knees and ankles. In a few hours spread to lower part of thighs. After two or three weeks, appeared on body, neck, face and shoulders. There was slight itching. Eruption tended to disappear and reappear on body and face. Any exertion aggravates eruption. Eruption papular and tubercular, the lesions being brownish or purplish at time of admission to hospital. The brownish spots mark the site of former lesions. Many pin-head points of effusion—some tending to confluence.

Patient was ordered to remain in bed, and given ℞: Pil. Ergotin gr. iii. t. i. d. Two days later the following were ordered: ℞: Potass. Acetat. Potass. Citrat. aa. ℥i. Aquæ ad .℥iv. m. Sig.: ʒi. t. i. d., and ℞: Tr. Ferri. Chlo., Ext. Ergot, fl., Glycerin. Aquæ aa. ℥i. m. ʒi. t. i. d. The eruption has disappeared, and pains are diminished.

The visiting assistant physicians recommend the use of spinal counter-irritation over nape of neck for the upper part of the body, and over the lumbo-sacral region for the lower part, in cases of intractable eczema, as first suggested by Crocker. Albolin, Mollin and Creolin, are recent additions to our skin pharmacopeia, but I am unable to specify their special indications.

An out-patient of the Skin and Cancer Hospital suffers from a rare disease; only one case is recorded similar to it. I have been unable to get the history as taken in the clinic, but will try and describe the case. The patient is a man about sixty years of age. Has had varicose veins of the legs for years. The skin of both knees is atrophied in the deeper layers, atrophy extending down the legs and up the thighs—anteriorly one-half or two-thirds of the thigh, and posteriorly about an equal distance on one, and up the natis on the other. In progression the skin first becomes purplish, then the hairs fall out. The skin gradually becomes thin and dry, the epidermic layers apparently unaffected, while the corium and sub-cutaneous tissue have lost in thickness. Where the process is complete, the skin is covered with a network of veins, slightly raised, and presenting a striking appearance. Disease, attributed to "nervous origin," through a history of definite shock or injury, was not obtained. The general health appears good.

Many applications are said to "pick out" the diseased tissue, as in epithelioma and lupus vulgaris, and leave uninjured the healthy parts. The following case seems to demonstrate such an action: Annie O'R., aged 24. Disease, lupus vulgaris, occupying right ear and region over parotid gland and extending toward temporal region. Began six years ago where ear had been pierced. Of exfoliative type, with well-marked, soft, tubercular points throughout. Emplastrum acid salicyl. 20 per cent first applied, having no marked effect. Four days later the following was applied: emplastrum ichthyolis, chrysorobin, acid salicyl. et zinci oxidi. Five days later many of the lupus tubercles were seen to have been "picked out," leaving round openings corresponding to tubercle's location. The healthy tissue unaffected. Simple ichthyol plaster then used for two weeks, probably to heal openings. Treatment then suspended six days, and the following next employed: ℞: ac. sal. gr. x, ungt. diachyli ℥iM. After four weeks, the comp. plaster of ichth. chryarob., ac. sal. and o. z. "renewed." Five days later, effect again seen markedly in the destruction of soft tubercles. This time there was slight irritation of the tissues.

The following shows how standard prescriptions may work badly:

J. S., aged 54. Seborrhoic eczema 6 years standing. ℞: resorcin ʒiii, sulph. sublimat. ℥i, ungt. zinc. ox. ℥ii. Returned with diffuse, erythematous, scaly ecxema over whole surface. An ointment of equal parts ungt. picis et zinc ox. and ungt. zinc ox. had perhaps a still more irritating effect, the entire skin becoming highly irritated. There is, however, a possibility that the last will act radically and cure the former disease.

M. B. HUTCHINS, M. D.

NEW YORK, April, 1889.

---

We have received a copy of "ON THE ROCK." A NOVEL. By Irene Farrar. James P. Harrison Company, Atlanta, Ga., 1889. That sterling journal, the *Southern Cultivator*, pays the following merited compliment to Atlanta's young and gifted authoress, which we reproduce and endorse: "Atlanta has reason to be proud of her new authoress. Not only Atlanta, but Georgia and the South. The new candidate for recognition is Miss Irene Farrar, of Atlanta, who has now in press at the publishing house of James P. Harrison Company, a novel entitled "On the Rock." It is printed on fine, heavy book paper, and its 293 pages reflect credit on the house. It is written in a style that interests and charms; easy, graceful and elevating. It is out of the beaten track, and yet is free from the sensational, slangy and wishy-washy mannerism that characterize many novels of the present period. It is natural and symmetrical. We predict for "On the Rock" an extraordinary sale, and for its gifted and brilliant authoress, unfading laurels in the Realm of Literature." For sale at all the bookstores, and by the J. P. Harrison Company, at $1.25 per copy. From present indications this edition will soon be exhausted. Get a copy while you can.

